# Isolated respiratory tract microorganisms and clinical characteristics in asthma exacerbation of obese patients: a multicenter study

**DOI:** 10.1186/s12890-024-02880-7

**Published:** 2024-02-02

**Authors:** Sojung Park, Yong Il Hwang, Sei Won Lee, Hyo-Jung Kim, Byung-Keun Kim, Jin Hwa Lee, Yon Ju Ryu, So Ri Kim, Jung Hyun Chang

**Affiliations:** 1 Division of Pulmonary and Critical Care Medicine, Department of Internal Medicine, Ewha Womans University Mokdong Hospital, Ewha Womans University College of Medicine, 1071 Anyangcheon-Ro, Yangcheon-gu, 07985 Seoul, Republic of Korea; 2 Division of Pulmonary, Allergy, and Critical Care Medicine, Department of Internal Medicine, Hallym University Sacred Heart Hospital, Hallym University College of Medicine, Anyang, Republic of Korea; 3 Department of Pulmonary and Critical Care Medicine, Asan Medical Center, University of Ulsan College of Medicine, Seoul, Republic of Korea; 4 Division of Pulmonology and Critical Care Medicine, Department of Internal Medicine, Inje University Haeundae Paik Hospital, Inje University College of Medicine, Busan, Republic of Korea; 5 Division of Pulmonology, Allergy and Critical Care Medicine, Department of Internal Medicine, Korea University Anam Hospital, Korea University College of Medicine, Seoul, Republic of Korea; 6 Division of Pulmonary and Critical Care Medicine, Department of Internal Medicine, Ewha Womans University Seoul Hospital, Ewha Womans University College of Medicine, Seoul, Republic of Korea; 7 Division of Respiratory Medicine and Allergy, Department of Internal Medicine, Research Center for Pulmonary Disorders, Jeonbuk National University Medical School, Jeonju, Republic of Korea

**Keywords:** Asthma, Bacteria, Exacerbation, Obesity, Virus

## Abstract

**Background:**

Viral infection is a risk factor for asthma exacerbation (AE). However, bacterial infections related to AE in adults are poorly known. On the other hand, obese patients with asthma have their own clinical and biological characteristics compared with non-obese patients.

**Methods:**

We investigated the differences in isolated pathogens for AE between obese and non-obese patients with asthma. We included 407 patients with AE from 24 medical centers in Korea. Microorganisms isolated from culture, RT-PCR or serologic tests using lower respiratory tract specimens were retrospectively investigated.

**Results:**

A total of 171 obese and 236 non-obese patients with asthma were included for analysis. Compared to non-obese patients, obese patients were associated with women (77.2% vs. 63.6%), never smoker (82.5% vs. 73.9%), shorter duration of asthma (7.9 ± 8.4 vs. 10.5 ± 10.1 years), less history of pulmonary tuberculosis (8.8% vs. 17.4%), and more comorbidity of allergic rhinitis (48.5% vs. 0.8%). Viral and/or bacterial infections were detected in 205 patients (50.4%) with AE. The numbers of patients with viral only, bacterial only, or both infections were 119, 49, and 37, respectively. The most commonly isolated bacterium was *Streptococcus pneumoniae*, followed by *Pseudomonas aeruginosa* and *Chlamydia pneumoniae*. Obese patients showed a lower incidence of *Chlamydia pneumoniae* infection. In the non-obese group, bacterial infection, especially *Chlamydia pneumoniae* infection, was significantly associated with the duration of systemic corticosteroid use (13.6 ± 19.8 vs. 9.7 ± 6.7 days, *p* = 0.049).

**Conclusion:**

Bacterial infection was associated with a longer period of corticosteroid use in the non-obese group. Acute *Chlamydia pneumoniae* infection was less associated with obese patients with AE. Further well-designed studies are needed to evaluate microorganisms and the efficacy of antibiotics in patients with AE.

## Introduction

Asthma is a chronic inflammatory airway disorder with various phenotypes, and obesity increases the risk of developing asthma 1.5 - to 3 - fold [1–3]. The association of asthma and obesity is now considered as a phenotype with its own clinical, biological and functional characteristics [4]. Obese patients with asthma often have impaired response to the inhaled corticosteroid (ICS)/long-acting beta-agonist (LABA) combination, and have worse asthma control with 4- to 6-fold higher risk of being hospitalized compared with non-obese patients with asthma [5, 6].

Approximately 60% of adult asthma exacerbations (AEs) are triggered by viral infection [7]. Human rhinovirus (HRV), respiratory syncytial virus (RSV), and influenza virus (IFV) are major causes of AEs [8]. However, few epidemiologic studies on bacterial infection in AE have been performed, and the potential role of bacterial infection in AE remains controversial. Chronic bacterial colonization is evident in the airway of patients with neutrophilic asthma, with *Haemophilus influenzae* (*H. influenzae)* being one of the most frequently isolated bacteria [9, 10]. Previous animal studies have shown that *H. influenzae* infection increases T helper 17-associated neutrophilic airway inflammation [11–13]. Bacterial community composition varies with disease features, steroid responses, and inflammatory phenotypes. Neutrophilic asthma is present in a greater proportion of obese than in non-obese patients with asthma [14, 15].

Bacteria in the lower airways are potential treatment targets, especially in steroid-resistant asthma. The aim of the present study was to investigate the differences in clinical characteristics and isolated pathogens of AEs between obese and non-obese patients and compare their treatment responses.

## Materials and methods

### Study population

We screened adult patients with AEs who were subjects for microbiological studies in 24 secondary or tertiary medical institutes in the Republic of Korea between January 2015 and December 2018. We included adult patients diagnosed with asthma at least 6 months before AEs regardless of treatment. AE was defined as an acute episode of progressive worsening of asthma symptoms requiring the use of oral/intravenous corticosteroids or more than doubling the dose of maintenance therapy. Of these, we included patients who had Gram staining and culture of sputum or endotracheal aspirates and multiplex reverse-transcription polymerase chain reaction (RT-PCR) for respiratory viruses of nasopharyngeal aspirates or lower respiratory tract specimens. During influenza season, antigen test or RT-PCR for influenza only, instead of RT-PCR for other viruses, was allowed. We excluded patients who had used antibiotics within 4 weeks before the AE episode, who had used 20 mg or more of prednisolone or an equivalent dose of another steroid, and who had used macrolide for more than 4 weeks.

Patients were classified into obese and non-obese groups, and their clinical characteristics, treatment response, and isolated pathogens were compared. Obesity was defined as a body mass index (BMI) ≥ 25.0 kg/m^2^ in accordance with the Asia-Pacific criteria of the World Health Organization guidelines [16].

Informed consents were waived because of the retrospective study design, and the study was approved by the institutional review board of the Ewha Womans University Mokdong hospital (EUMC 2019-06-017).

### Assessment

The present study investigated the clinical characteristics and isolated pathogens of AEs and compared them between the obese and non-obese groups. Demographic and clinical information of patients were retrospectively collected from electronic medical records. The following variables were assessed: age, sex, BMI, smoking history, comorbidities, treatment regimen for asthma maintenance therapy at the time of AE, and the level of asthma control within 3 months before the episode of AE. Diagnostic criteria for asthma and evaluation of the level of asthma control followed the GINA guideline 2018 [17]. Comorbidities were investigated through history taking from the patient or review of past medical history at the time of AE. Comorbidity was defined as a condition that the patient currently has or is currently receiving repeated treatment for, except history of tuberculosis. We also included newly diagnosed comorbidities during AE. Symptoms and severity of AE, duration of corticosteroids use, antibiotics and treatment response were also evaluated.

### Microbiological evaluation

Viruses and bacteria confirmed by microbiological evaluation at the time of AE diagnosis were investigated. The specific diagnostic kits for the detection of pathogens were different among institutes. Microbiological studies included the following: sputum or endotracheal aspirates, or bronchoalveolar lavage (BAL) fluid for Gram staining and culture; sputum or endotracheal aspirates, or BAL fluid for RT-PCR and/or serology test for *Mycoplasma pneumoniae, Chlamydia pneumoniae* (*C. pneumoniae*), *Legionella pneumophila*, and *Bordetella pertussis*; nasopharyngeal aspirates, sputum, endotracheal aspirates, or BAL fluid for multiplex RT-PCR for IFV A and B, RSV, HRV, parainfluenza virus 1 to 4, adenovirus, human coronavirus 229E and OC43, human metapneumovirus, enterovirus, and bocavirus.

### Statistical analysis

Pearson chi-square test or Fisher’s exact test was used to compare categorical variables, and Student t-test or Mann-Whitney test was used to compare continuous variables. All tests of significance were two-sided, and differences among groups were considered significant when the *p*-value was < 0.05. All statistical analyses were performed with SPSS software version 22.0 (IBM Corporation, Armonk, NY, USA).

## Results

### Baseline characteristics

A total of 407 patients, 171 (42.0%) obese and 236 (58.0%) non-obese, were included in the present study. Table 1 shows the demographics and clinical characteristics of the patients. The mean age was 66.4 ± 17.4 years; 282 (69.3%) were women. The obese group included significantly more proportion of never smokers compared with the non-obese group (82.5% vs. 73.9%, *p* = 0.026). There were significant differences in sex, the duration of asthma, past history of pulmonary tuberculosis, and comorbidity of allergic rhinitis between the two groups.

**Table 1:** Baseline characteristics of patients with asthma exacerbation

	Total	No obesity	Obesity	*P*
	*n* = 407	*n* = 236	*n* = 171	
Age, years	66.4 ± 17.4	66.6 ± 17.9	66.2 ± 16.6	0.797
Sex, women	282 (69.3)	150 (63.6)	132 (77.2)	0.003
BMI, kg/m^2^	24.8 ± 4.6	21.7 ± 2.2	29.0 ± 3.5	< 0.001
Smoking history				0.026
Ex-smoker	51 (12.6)	30 (12.8)	21 (12.3)	
Current smoker	40 (9.9)	31 (13.2)	9 (5.3)	
Never-smoker	314 (77.5)	173 (73.9)	141 (82.5)	
Disease period, year	9.4 ± 9.5	10.5 ± 10.1	7.9 ± 8.4	0.009
Underlying disease				
Diabetes mellitus	91 (22.4)	52 (22.0)	39 (22.8)	0.853
Allergic rhinitis	85 (20.9)	2 (0.8)	83 (48.5)	< 0.001
History of TB	56 (13.8)	41 (17.4)	15 (8.8)	0.013
ILD	55 (13.5)	29 (12.3)	26 (15.2)	0.396
Liver cirrhosis	49 (12.0)	34 (14.4)	15 (8.8)	0.085
Sinusitis	31 (7.6)	20 (8.5)	11 (6.4)	0.443
CHF	16 (3.9)	7 (3.0)	9 (5.3)	0.239
CKD	15 (3.7)	9 (3.8)	6 (3.5)	0.872
Food allergy	11 (2.7)	6 (2.5)	5 (2.9)	0.815
Bronchiectasis	2 (0.5)	1 (0.4)	1 (0.6)	0.819

Data are shown as n (%) per each group or means ± standard deviation

BMI, body mass index; TB, tuberculosis; ILD, interstitial lung disease; CHF, congestive heart failure; CKD, chronic kidney disease

### Level of disease control

There was no significant difference in usual maintenance treatment between the two groups (Table 2). An ICS/LABA combination therapy was the most commonly prescribed medication (48.8%), followed by an ICS/LABA/long-acting muscarinic antagonist combination therapy (14.7%). A total of 27.8% of patients were not receiving maintenance treatment at the time of AE. There was no significant difference in the level of asthma control between the two groups, although, more patients in the obese group had uncontrolled asthma (41.4% vs. 30.5%, *p* = 0.077).

**Table 2:** Prescribed medications for asthma maintenance therapy and the level of asthma control at the time of asthma exacerbation

	Total	No obesity	Obesity	*P*
	*n* = 381^*^	*n* = 216	*n* = 165	
Prescribed respiratory medicines				
ICS + LABA	186 (48.8)	102 (47.2)	84 (50.9)	0.476
ICS	26 (6.8)	14 (6.5)	12 (7.3)	0.762
LAMA	4 (1.0)	2 (0.9)	2 (1.2)	0.786
LAMA + LABA	3 (0.8)	3 (1.4)	0	0.262
ICS + LABA + LAMA	56 (14.7)	37 (17.1)	19 (11.5)	0.125
No treatment	106 (27.8)	58 (26.9)	48 (29.1)	0.629
LTRA	185 (48.8)	103 (47.7)	82 (50.3)	0.613
Oral steroid	46 (12.1)	28 (13.0)	18 (11.0)	0.571
Biologics	6 (1.6)	5 (2.3)	1 (0.6)	0.243
Level of controls^*,†^				0.208
Uncontrolled	85/239 (35.6)	39/128 (30.5)	46/111 (41.4)	0.077
Partly controlled	101/239 (42.3)	58/128 (45.3)	43/111 (38.7)	0.305
Well controlled	53/239 (22.2)	31/128 (24.2)	22/111 (19.8)	0.414

Data are shown as n (%) per each group

*Not all the medication records or the level of asthma control were investigated because one of 24 medical institutions only allowed to a portion of their medical records. The denominator refers to the number of patients investigated

^†^The level of asthma control within 3 months before the episode of AE

ICS, inhaled corticosteroid; LABA, long-acting beta-agonist; LAMA, long-acting muscarinic antagonist; LTRA, leukotriene receptor antagonist

### Isolated pathogens and empirical antimicrobial therapy

**Fig. 1:**
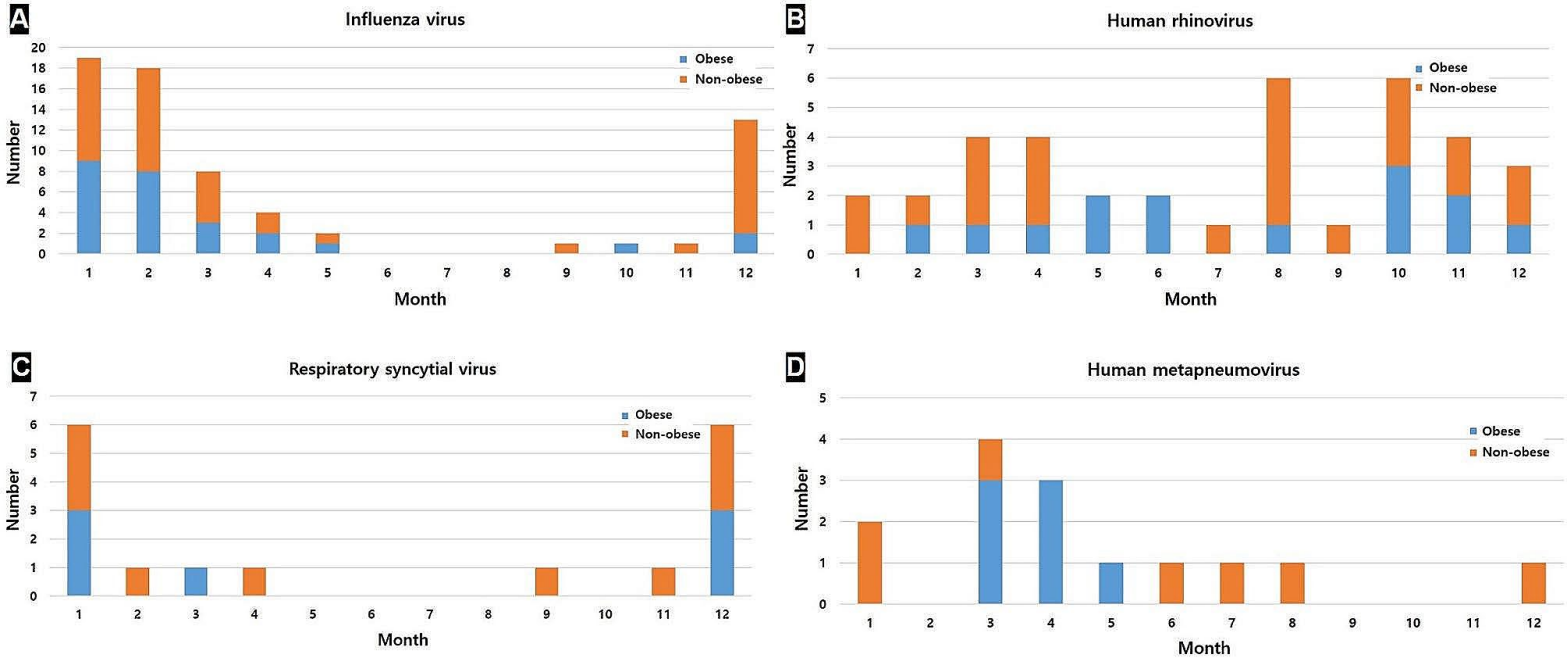
Seasonal frequency of viruses. Influenza virus (**A**), human rhinovirus (**B**), respiratory syncytial virus (**C**), and human metapneumovirus (**D**)

Viral or bacterial infection was detected in 205 (50.4%) patients (Table 3). The numbers of patients with viral only, bacterial only, or both infections were 119, 49, and 37, respectively. The most commonly isolated virus was IFV (*n* = 67), followed by HRV (*n* = 37) and RSV (*n* = 17). There was no significant difference in the incidence of viral infection between the two groups. IFV and RSV infections showed a peak prevalence in winter, while HRV infections seemed to occur throughout the year (Fig. 1).

**Table 3:** Isolated pathogens during asthma exacerbation

	Total	No obesity	Obesity	*P*
	*n* = 407^*^	*n* = 236	*n* = 171	
**Virus**				
Influenza	67/407 (16.5)	41/236 (17.4)	26/171 (15.2)	0.560
Human rhinovirus	37/328 (11.3)	23/196 (11.7)	14/132 (10.6)	0.751
RSV	17/326 (5.2)	10/194 (5.2)	7/132 (5.3)	0.953
Metapneumovirus	14/326 (4.3)	7/195 (3.6)	7/131 (5.3)	0.444
Coronavirus	12/328 (3.7)	9/196 (4.6)	3/132 (2.3)	0.374
Parainfluenza	10/328 (3.0)	6/196 (3.1)	4/132 (3.0)	1.000
Adenovirus	3/328 (0.9)	1/196 (0.5)	2/132 (1.5)	0.567
Enterovirus	1/264 (0.4)	0	1/105 (1.0)	0.398
**Bacteria**				
*S. pneumoniae*	25/407 (6.1)	15/236 (6.4)	10/171 (5.8)	0.833
*P. aeruginosa*	17/407 (4.2)	12/236 (5.1)	5/171 (2.9)	0.282
*C. pneumoniae*	10/217 (4.6)	9/119 (7.6)	1/98 (1.0)	0.024
*K. pneumonia*	9/407 (2.2)	5/236 (2.1)	4/171 (2.3)	1.000
*M. pneumoniae*	9/254 (3.5)	4/138 (2.9)	5/116 (4.3)	0.736
*H. influenzae*	8/407 (2.0)	4/236 (1.7)	4/171 (2.3)	0.725
*E. coli*	6/407 (1.5)	4/236 (1.7)	2/171 (1.2)	1.000
MRSA	4/407 (1.0)	2/236 (0.8)	2/171 (1.2)	1.000
*M. catarrhalis*	3/407 (0.7)	1/236 (0.4)	2/171 (1.2)	0.575
MSSA	2/407 (0.5)	0	2/171 (1.2)	0.176
*S. maltophilia*	2/407 (0.5)	2/236 (0.8)	0	0.512
*B. pertussis*	1/126 (0.8)	0	1/54 (1.9)	0.429

Data are shown as n (%) per each group

^*^The denominator refers to the number of patients investigated

RSV, respiratory syncytial virus; *S. pneumoniae*, *Streptococcus pneumoniae*; *P. aeruginosa*, *Pseudomonas aeruginosa*; *C. pneumoniae*, *Chlamydia pneumoniae*; *K. pneumoniae*, *Klebsiella pneumoniae*; *M. pneumoniae*, *Mycoplasma pneumoniae*; *H. influenzae*, *Haemophilus influenzae*; *E. coli*, *Escherichia coli*; MRSA, methicillin-resistant *Staphylococcus aureus*; *M. catarrhalis*, *Moraxella catarrhalis*; MSSA, methicillin-susceptive *Staphylococcus aureus*; *S. maltophilia*, *Stenotrophomonas maltophilia*; *B. pertussis*, *Bordetella pertussis*

The dominantly isolated bacteria were *Streptococcus pneumoniae* (*S. pneumoniae*, *n* = 25), *Pseudomonas aeruginosa* (*n* = 17), and *C. pneumoniae* (*n* = 10). Nine out of 10 patients with *Chlamydia* infection showed positive IgM test results and the remaining one showed a positive RT-PCR test result. Compared with the non-obese group, the obese group showed a lower incidence of *C. pneumoniae* infection (1.0% vs. 7.6%, *p* = 0.024). A total of 337 patients (88.9%, Table 4) received antibiotics; the most commonly prescribed antibiotics was beta-lactam (227/337, 67.4%), followed by quinolone (77/337, 22.8%), and macrolide (20/337, 5.9%).

**Table 4:** Treatment and healthcare utilization in patients who experienced asthma exacerbation

	Total	No obesity	Obesity	*P*
	*n* = 379^*^	*n* = 216	*n* = 163	
Corticosteroid use	341 (90.0)	191 (88.4)	150 (92.0)	0.048
Duration of corticosteroid use, days	11.5 ± 11.5	10.4 ± 10.5	12.8 ± 12.6	0.066
Admission rate	366 (96.6)	208 (96.3)	158 (96.9)	0.736
ICU admission rate	35 (9.2)	23 (10.6)	12 (7.4)	0.274
Duration of hospitalization, days	9.7 ± 7.9	9.7 ± 8.3	9.7 ± 7.4	0.940
Duration of exacerbation, days	11.0 ± 8.8	10.4 ± 8.4	11.7 ± 9.2	0.159
Antibiotics	337 (88.9)	190 (88.0)	147 (90.2)	0.733
Beta-lactam	227 (67.4)	127 (66.8)	100 (68.0)	
Quinolone	77 (22.8)	40 (21.1)	37 (25.2)	
Macrolide	20 (5.9)	13 (6.8)	7 (4.8)	
Miscellaneous	13 (3.9)	10 (5.3)	3 (2.0)	

Data are shown as n (%) per each group or means ± standard deviation

*Not all the records regarding medication or healthcare use were investigated because one of 24 medical institutions only allowed to a portion of their medical records. The denominator refers to the number of patients investigated

ICU, intensive care unit

### Treatment outcomes of asthma exacerbations

Eight patients in the obese group and 20 patients in the non-obese group had missing data regarding steroid and healthcare use. Except these, data of 379 patients were analyzed regarding treatment outcomes of AE. A total of 341 (341/379, 90.0%) patients received systemic corticosteroids for treatment of AE . There were no significant differences in admission rate, intensive care unit admission rate, length of hospital stay, or AE duration between the two groups. Significantly more patients in the obese group had received systemic corticosteroids (92.0% vs. 88.4%, *p* = 0.048) with a tendency for a longer period of corticosteroid use (12.8 ± 12.6 vs. 10.4 ± 10.5 days, *p* = 0.066) compared with the non-obese group (Table 4). In subgroup analysis with the obese group, there were no significant differences in treatment outcomes depending on viral or bacterial infection (Table 5). However, in the non-obese group, bacterial infection was associated with a longer period of corticosteroid use (13.6 ± 19.8 vs. 9.7 ± 6.7 days, *p* = 0.049). In addition, infection with *C. pneumoniae* was associated with longer AE duration (22.7 ± 15.9 vs. 9.9 ± 7.5 days, *p* < 0.001) and longer corticosteroid use (25.0 ± 39.2 vs. 9.8 ± 6.7 days, *p* < 0.001) in the non-obese group.

**Table 5:** Severity of asthma exacerbation depending on obesity and infection

	No obesity				Obesity			
	Total	Infection	No infection	*P*	Total	Infection	No infection	*P*
	*n* = 216	*n* = 104	*n* = 112		*n* = 163	*n* = 76	*n* = 87	*n* = 163
Admission rate	208 (96.3)	98 (94.2)	110 (98.2)	0.158	158 (96.9)	76 (100.0)	82 (94.3)	0.061
ICU admission rate	23 (10.6)	10 (8.9)	13 (12.5)	0.395	12 (7.4)	6 (7.9)	6 (6.9)	0.808
Duration of steroid use, days	10.4 ± 10.5	11.0 ± 13.1	9.9 ± 7.0	0.456	12.8 ± 12.6	12.7 ± 9.6	12.8 ± 14.8	0.983
Duration of exacerbation, days	10.4 ± 8.4	11.2 ± 9.9	9.6 ± 6.4	0.173	11.7 ± 9.2	11.9 ± 8.1	11.5 ± 10.2	0.768
	Total *n* = 216	Bacterial infection *n* = 45	No infection *n* = 171	*P*	Total *n* = 163	Bacterial infection *n* = 31	No infection *n* = 132	*P*
Duration of steroid use, days	10.4 ± 10.5	13.6 ± 19.8	9.7 ± 6.7	0.049	12.8 ± 12.6	14.6 ± 8.8	12.3 ± 13.3	0.392
Duration of exacerbation, days	10.4 ± 8.4	12.5 ± 10.0	9.9 ± 7.8	0.061	11.7 ± 9.2	12.0 ± 7.1	11.7 ± 9.7	0.868
	Total *n* = 216	*Chlamydia* infection *n* = 9	No infection *n* = 207	*P*	Total *n* = 163	*Chlamydia* infection *n* = 1	No infection *n* = 162	*P*
Duration of steroid use, days	10.4 ± 10.5	25.0 ± 39.2	9.8 ± 6.7	< 0.001	12.8 ± 12.6	22	12.7 ± 12.6	0.463
Duration of exacerbation, days	10.4 ± 8.4	22.7 ± 15.9	9.9 ± 7.5	< 0.001	11.7 ± 9.2	7	11.7 ± 9.2	0.610

Data are shown as n (%) per each group or means ± standard deviation

ICU, intensive care unit

## Discussion

In the present study, we found that bacterial infection was identified in 21.1% of all patients with AE. Obese patients with AE used more systemic corticosteroids and had less *C*. *pneumoniae* infection compared with non-obese patients. Bacterial infection, especially *C. pneumoniae* infection, was associated with longer periods of corticosteroid use in the non-obese group.

Consistent with previous reports, HRV, IFV, and RSV were the most commonly isolated viruses in the present study [8]. Johnston et al. reported that oseltamivir decreases the frequency and symptom severity of AE in children [18]. However, the identification of viral pathogens in AE is of limited value in clinical practice because antiviral treatment is limited in many cases except IFV infection. The present study showed a high incidence of typical respiratory pathogens, such as *S. pneumoniae* and *Pseudomonas aeruginosa*, as well as atypical pathogens such as *C. pneumoniae* and *Mycoplasma pneumoniae*. Iikura et al. reported that typical pathogens were commonly isolated in Japanese patients with AE [19]. In addition, upper airway detection of *S. pneumoniae* during HRV infection is associated with a prevalence of moderate AE [20]. In the present study, bacterial infection, especially *C. pneumoniae* infection, was associated with longer AE duration and longer periods of corticosteroid use in the non-obese group. Several studies have reported that acute or chronic infection of *C. pneumoniae* is associated with severe asthma [21–23]. The present study showed that almost all patients with isolated *C. pneumoniae* showed positive IgM test results, which indicates an acute infection. Although the reason for the higher incidence of *Chlamydia* infection in non-obese patients is unclear, identification of *C. pneumoniae* as an acute infectious pathogen as well as colonization might be important in uncontrolled asthma or AE.

There are few epidemiological studies on bacterial infection in AE. Previous clinical trials excluded patients who had received antibiotics at the time of enrollment, those with smoking history, or those with comorbid chronic obstructive pulmonary disease. These patients are likely to benefit from antibiotics, and, some clinicians use empirical antibiotics during AE in clinical practice. A Cochrane review reported that use of antibiotics in patients with AE was associated with longer symptom-free days, shorter periods of AE and higher peak expiratory flow rate [24]. Because little is known about the most appropriate empiric antibiotic or duration of its use, epidemiological studies on bacterial infection in AE is needed to prevent inappropriate use or overuse of antibiotics.

Scott et al. have shown that neutrophilic airway inflammation improves with weight loss in women [15, 25]. In addition, there is increasing evidence that asthma is associated with changes in the airway microbiome, which may be altered in obese patients. A recent study including patients with severe asthma showed that obese patients had significantly abundant all taxa and fewer eosinophils in bronchial brushings compared with non-obese patients [26]. These results may suggest that obesity or altered microbiome or both is associated with less eosinophilic airway inflammation. Impaired response to corticosteroids in obesity might result from its altered pathogenesis, which is related to chronic low-grade inflammation affecting the adipose tissue but might also be associated with bacterial burden [27, 28]. Because antibiotics may induce the alteration of microbiome composition and antibiotic resistant pathogens, antibiotics should be used cautiously. A total of 88.9% of patients in the present study were prescribed antibiotics, which was higher than we expected. We could not determine whether the patients who received antibiotics had clear signs, symptoms or laboratory test results suggestive of bacterial infection. Isolated microbial data in AE may guide to appropriate use of antibiotics and prevent overuse of antibiotics.

This study has several limitations. Firstly, because only patients with RT-PCR for viruses and culture for bacteria were included,. relatively small number of patients were included in the present study although we included patients from 24 medical institutes across Republic of Korea. Also, patients with severe symptoms requiring hospitalization or those with old age and underlying disease might be preferentially selected. This might have caused a selection bias that excluded younger patients with increased T helper 2-type allergic inflammation. Second, we did not compare the patients with AE with those with stable asthma or with healthy individuals; therefore, the findings cannot be distinguished from colonization. Therefore, further well-designed prospective comparative studies are warranted. Third, antibiotic susceptibility test results for the isolated bacteria could not be found, so it was not possible to evaluate the impact of the susceptibility test results on treatment outcomes. Fourth, we could not correct for differences among institutions because a large number of medical institutions participated in the study and a large difference in the number of patients registered at each institution. Fifth, we could not perform a trend test which determines the seasonality of viral infections each year. Although viral seasonality in the present study was consistent with the results of other nation-wide study conducted in Korea, it is necessary to collect and investigate data over a longer period of time [29].

## Conclusions

Bacteria were isolated in 21.1% of patients with AE. Bacterial infection, especially *C. pneumoniae* infection, was associated with a longer period of corticosteroid use in the non-obese group. *Chlamydia pneumoniae* was less isolated with obese patients with AE. Obese patients with AE required more systemic corticosteroids with a tendency for a longer period of corticosteroid use compared with non-obese patients. Further well-designed studies are needed to evaluate microorganisms and the efficacy of antibiotics in patients with AE.

## Data Availability

The datasets generated and analyzed for this study are not publicly available but are available from the corresponding author upon reasonable request.

## Abbreviations

ICS: Inhaled corticosteroid
LABA: long-acting beta-agonist
AE: Asthma exacerbation
HRV: Human rhinovirus
RSV: Respiratory syncytial virus
IFV: Influenza virus
*H. influenzae*: *Haemophilus influenzae*
RT-PCR: Reverse-transcription polymerase chain reaction
BMI: Body mass index
BAL: Bronchoalveolar lavage
*C. pneumoniae*: *Chlamydia pneumoniae*
*S. pneumoniae*: *Streptococcus pneumoniae*
